# Regulation of Inflammatory Response in Human Osteoarthritic Chondrocytes by Novel Herbal Small Molecules

**DOI:** 10.3390/ijms20225745

**Published:** 2019-11-15

**Authors:** Reihane Ziadlou, Andrea Barbero, Martin J. Stoddart, Michael Wirth, Zhen Li, Ivan Martin, Xin-luan Wang, Ling Qin, Mauro Alini, Sibylle Grad

**Affiliations:** 1 AO Research Institute Davos, 7270 Davos Platz, Switzerland; reihane.ziadlou@aofoundation.org (R.Z.); martin.stoddart@aofoundation.org (M.J.S.); michael.a.wirth@bluewin.ch (M.W.); zhen.li@aofoundation.org (Z.L.); mauro.alini@aofoundation.org (M.A.); 2 Department of Biomedical Engineering, University of Basel, 4123 Allschwil, Switzerland; andrea.barbero@usb.ch (A.B.); ivan.martin@usb.ch (I.M.); 3 Department of Biomedicine, University Hospital Basel, University of Basel, 4001 Basel, Switzerland; 4 Translational Medicine R&D Center, Shenzhen Institutes of Advanced Technology, Chinese Academy of Sciences, Shenzhen 518057, China; xl.wang@siat.ac.cn (X.-l.W.); lingqin@cuhk.edu.hk (L.Q.); 5 Department of Orthopaedics & Traumatology, the Chinese University of Hong Kong, Hong Kong SAR, China; 6 Department of Health Sciences and Technology, ETH Zürich, 8092 Zürich, Switzerland

**Keywords:** osteoarthritis, traditional Chinese medicine compounds, osteoarthritic chondrocytes, anti-inflammatory effects, anabolic, catabolic

## Abstract

In this study, 34 Traditional Chinese Medicine (TCM) compounds were screened for potential anabolic and anti-inflammatory properties on *human* osteoarthritic (OA) chondrocytes. The anabolic effects were assessed by measuring the glycosaminoglycan (GAG) relative to the DNA content using a 3D pellet culture model. The most chondrogenic compounds were tested in an inflammatory model consisting of 3 days of treatment with cytokines (IL-1β/TNF-α) with or without supplementation of TCM compounds. The anti-inflammatory effects were assessed transcriptionally, biochemically and histologically. From the 34 compounds, Vanilic acid (VA), Epimedin A (Epi A) and C (Epi C), 2′′-O-rhamnosylicariside II (2-O-rhs II), Icariin, Psoralidin (PS), Protocatechuicaldehyde (PCA), 4-Hydroxybenzoic acid (4-HBA) and 5-Hydroxymethylfurfural (5-HMF) showed the most profound anabolic effects. After induction of inflammation, pro-inflammatory and catabolic genes were upregulated, and GAG/DNA was decreased. VA, Epi C, PS, PCA, 4-HBA and 5-HMF exhibited anti-catabolic and anti-inflammatory effects and prevented the up-regulation of pro-inflammatory markers including metalloproteinases and cyclooxygenase 2. After two weeks of treatment with TCM compounds, the GAG/DNA ratio was restored compared with the negative control group. Immunohistochemistry and Safranin-O staining confirmed superior amounts of cartilaginous matrix in treated pellets. In conclusion, VA, Epi C, PS, PCA, 4-HBA and 5-HMF showed promising anabolic and anti-inflammatory effects.

## 1. Introduction

Occurrence of age-related musculoskeletal disorders is increasing, with osteoarthritis (OA) being the most prevalent joint disorder. Approximately 20% of the adult population in Europe and the US is expected to be afflicted with OA by 2030 [1,2,3], resulting in excessive economic burdens on healthcare systems. 

Development of OA results from a combination of genetic and environmental factors including obesity, ageing, acute joint trauma and inflammation, which cause an imbalance between chondrocyte anabolism and catabolism and primarily affect articular hyaline cartilage in load bearing joints [4,5,6,7,8]. Furthermore, OA can increase the risk of onset of other chronic diseases, making prevention and early effective treatment of OA a relevant research focus [9]. The avascular nature of cartilage, combined with low cell density and slow matrix turnover rate, results in a lack of effective methods to prevent or treat OA [7]. If conventional treatment such as physical therapy or weight reduction fails to alleviate symptoms, simple analgesics are initially recommended (e.g., acetaminophen). Additionally, non-steroidal anti-inflammatory drugs (NSAIDs), including specific or non-specific cyclooxygenase 2 (COX-2) inhibitors, are used frequently [10,11,12]. However, these systemic treatments are often insufficient and bear the risks of gastrointestinal, renal or cardiovascular side effects [13,14,15]. Alternative anti-inflammatory treatments include the inhibition of inducible nitric oxide synthase (iNOS) or nuclear factor kappa B (NFκB) signaling [16]. Furthermore, anti-cytokine therapy, such as anti-tumor necrosis factor (TNF)-α antibodies, can potentially suppress inflammation and prevent cartilage degradation in OA patients [17]. However, in addition to TNF-α, other pro-inflammatory cytokines such as interleukin (IL)-1, are also involved in the degenerative process [18]. Anti-matrix metalloproteinase (MMP) therapy faces a similar concern, as multiple enzymes contribute to the complex breakdown mechanism of the extracellular matrix. When the disease is progressing (Stage 3), intra-articular injection of hyaluronic acid, glucosamine or corticosteroids can be considered [19]. Though effects are often initially satisfactory, these injections only achieve short-term pain relief, and eventually surgical joint replacement is one of the last options remaining in the Stage 4 of OA [20]. Joint replacement is a highly invasive procedure with an extended rehabilitation period and limited options for revision when necessary [21]. Alternatively, arthroscopic lavage and debridement have been applied in OA patients with variable outcomes [22]. Since conventional treatment approaches aim at symptoms relief and do not address the catabolic component of the destructive OA disease [23], the development of innovative, disease modifying and low risk treatments for OA is an unmet clinical need.

Several plant-derived compounds have been identified that have the potential to inhibit NFκB signaling and inflammation [24,25,26,27,28]. Well known plant-derived compounds used for these purposes are curcumin and resveratrol [29]. In addition, extracts of Traditional Chinese Medicine (TCM) herbs have been investigated for their anti-inflammatory effects. For example, *Caesalpinia sappan* derived extracts were shown to suppress nitric oxide synthesis in osteoarthritic chondrocytes by down-regulating the iNOS mRNA expression. It was concluded that blockage of IL-1 induced NFκB signaling and its down-stream pro-inflammatory targets by *Caesalpinia sappan* extracts may counteract cartilage breakdown in OA [18]. Similarly, Honokiol, a low molecular weight natural product isolated from *Magnolia officinalis*, was able to prevent IL-1 induced inflammatory reactions and cartilage matrix breakdown in *human* OA chondrocytes [30]. 

Therapy with herbal Fufang is popular in TCM for prevention and treatment of osteoporosis and related bone diseases. Xianlinggubao formula (XLGB) was formed based on modification of the empirical “Miao minority” medicine, which was commonly used to tone the “kidney system” and nourish bones [31]. XLGB capsule was officially approved by the Chinese State Food and Drug Administration (cFDA) as the over-the-counter drug for treatment of osteoporosis [32], aseptic osteonecrosis [33], osteoarthritis [34] and bone fractures [32]. XLGB is composed of six kinds of herbs containing various compounds [32,33]. A total of 118 compounds were identified from XLGB extract [35]. Some of them, e.g., Icariin, had previously demonstrated extensive bioactivity and anti-inflammatory activity and were used as bioactive factors in tissue engineering for cartilage defect repair [36,37].

We hypothesized that, among the various compounds identified in TCM herbal extracts, there are distinct molecules with potent chondrogenic and anti-inflammatory effects. However, no comprehensive and systematic direct comparison of diverse TCM molecules in terms of combined chondrogenic and anti-inflammatory properties has been performed. The current study assessed the anabolic and anti-inflammatory effects of 34 relevant TCM compounds from XLGB on *human* osteoarthritic chondrocytes. The aim was to identify the most potent compounds in terms of cartilage matrix synthesis and counteraction of inflammatory responses. 

## 2. Results

### 2.1. Toxicity Assay for the Compounds on Human Osteoarthritic Chondrocytes

Cytotoxicity assay of monolayer cultures showed that after 48 h of treatment with TCM compounds (1 µM, 10 µM, 25 µM, 50 µM), more than 75% of the cells were viable in all treatment groups. Furthermore, in different concentrations of dimethyl sulfoxide (DMSO) (0.01%, 0.1%, 0.25%, 0.5% *v/v*) as control vehicle group the numbers of viable cells were consistent. So, DMSO did not show any toxic effect on the cells in these concentrations. Furthermore, the viability of the cells in the un-treated control group was not different from the control vehicle group. All data are normalized to the control vehicle group (Table 1). 

### 2.2. Anabolic Effects of Traditional Chinese Medicine (TCM) Compounds on Glycosaminoglycan Production

**Figure 1:**
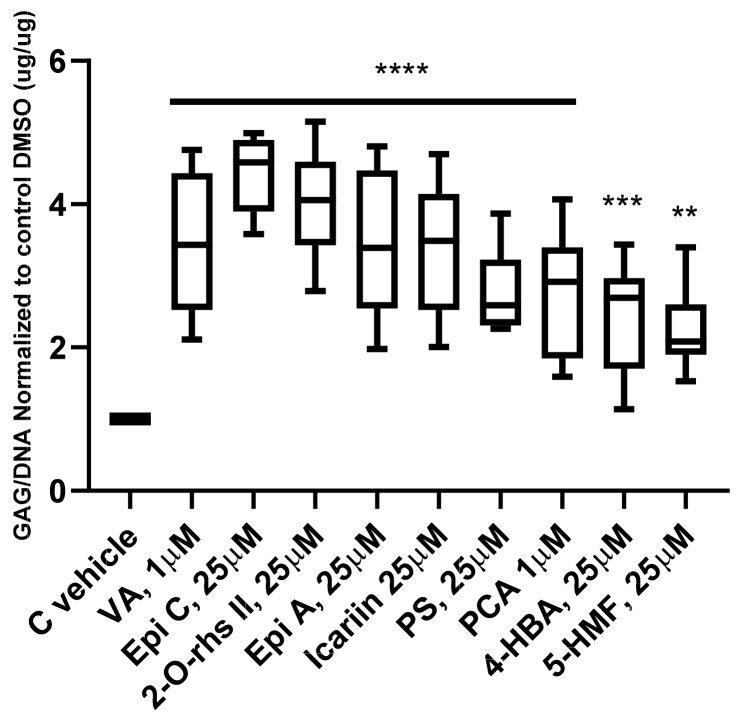
Glycosaminoglycan (GAG) production of 3D *human* osteoarthritic chondrocyte pellet cultures after two weeks in chondro-permissive medium supplemented with Traditional Chinese Medicine (TCM) compounds. Glycosaminoglycan content was normalized to the amount of DNA. The most effective doses of compounds which could promote GAG production versus control vehicle (C vehicle) group in 3/3 donors are shown; for each donor three experimental replicates were analyzed. For statistical analysis using Graphpad Prism, one-way analysis of variance (ANOVA) followed by Dunnett’s post hoc test (multiple comparison) was applied. ** *p* < 0.001, *** *p* < 0.0005, **** *p* < 0.0001 versus control vehicle. Vanilic acid (VA), Epimedin A (Epi A) and C (Epi C), 2′′-O-rhamnosylicariside II (2-O-rhs II), Icariin, Psoralidin (PS), Protocatechuicaldehyde (PCA), 4-Hydroxybenzoic acid (4-HBA), 5-Hydroxymethylfurfural (5-HMF).

To determine the anabolic effect of the TCM compounds, all 34 compounds were tested in 3D pellet culture in four different concentrations (1 µM, 10 µM, 25 µM, 50 µM). *Human* OA chondrocytes from three donors were cultured in chondro-permissive medium with supplementation of compounds or with DMSO (0.01%, 0.1%, 0.25%, 0.5% *v/v*) (control vehicle group) for two weeks. Nine compounds, namely Vanilic acid (VA), Epimedin A (Epi A) and C (Epi C), 2′′-O-rhamnosylicariside II (2-O-rhs II), Psoralidin (PS), Icariin, Protocatechuicaldehyde (PCA), 4-Hydroxybenzoic acid (4-HBA) and 5-Hydroxymethylfurfural (5-HMF) significantly increased the GAG/DNA ratio compared to the control vehicle group in all three donors. All data are normalized to their respective control vehicle groups, and the compounds which were effective in all the different concentrations are shown in Figure 1 in their most effective dose. The complete data set from all compounds tested in four concentrations is shown in Supplementary Table S1. Epi A, Epi C, 2-O-rhs II and Icariin are all extracts of *Epimedium* medicinal herb with similar chemical structures and positive anabolic effects. Since Epi C showed the highest amount of matrix production among these four compounds, it was chosen as a representative of *Epimedium* herb extract for use in further experiments. Furthermore, VA, PS, PCA, 4-HBA and 5-HMF from the first screening in their most effective dose were selected for further studies in the inflammatory models.

### 2.3. Effects of TCM Compounds on Pro-Inflammatory and Pro-Catabolic Gene Expression under Inflammatory Conditions

**Figure 2:**
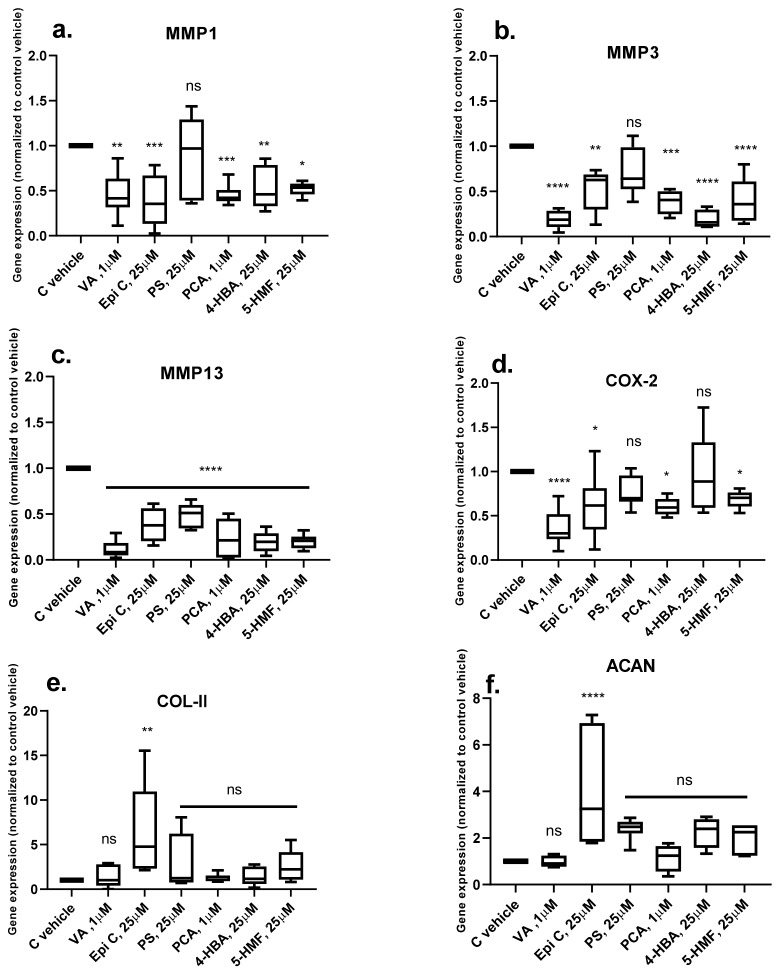
Transcriptional level of catabolic genes matrix metalloproteinase (MMP) **a**) *MMP1* **b**) *MMP3* **c**) *MMP13*, pro-inflammatory gene **d**) cyclooxygenase 2 *(COX-2)* and anabolic marker genes **e**) collagen type II (*COL*-*II)* and **f**) aggrecan (*ACAN)* in Phase II (short term pellets) simultaneously treated with TCM compounds and IL-1β/TNF-α versus control vehicle (C vehicle) group in *human* OA chondrocytes. *n* = 3; n indicates the number of *human* OA donors; for each donor three experimental replicates were analyzed. Data are normalized to the levels of control groups. For statistical analysis using Graphpad Prism, one-way analysis of variance (ANOVA) followed by Dunnett’s post hoc test (multiple comparisons) was applied. * *p* < 0.01, ** *p* < 0.001, *** *p* < 0.0005, **** *p* < 0.0001, ns (non-significant).

After one week of cartilage generation (short-term) in phase I, the pro-catabolic and pro-inflammatory genes in the pellets treated with IL-1β/TNF-α in phase II were significantly increased. To investigate the inhibitory effects of the selected compounds in phase II, compounds in their most effective dose were simultaneously added with inflammatory cytokines to the chondro-permissive culture media. We observed that after treatment with VA, Epi C, PS, PCA, 4-HBA and 5-HMF, the catabolic marker gene matrix metalloproteinase 13 (*MMP13)* was significantly downregulated in all treatment groups. In the groups treated with VA, Epi C, PCA, 4-HBA and 5-HMF, other catabolic marker genes matrix metalloproteinase 1, 3 (*MMP1, MMP3)* were also significantly inhibited compared with the control DMSO group. Furthermore, *COX-2* was significantly downregulated in all the treatment groups except for PS and 4-HBA. In pellets treated with Epi C, in addition to downregulation of catabolic marker genes, the anabolic marker genes collagen type II (*COL-II)* and aggrecan (*ACAN)* were significantly upregulated (Figure 2a–f). 

### 2.4. Effect of TCM Compounds on Matrix Production under Inflammatory Conditions

**Figure 3:**
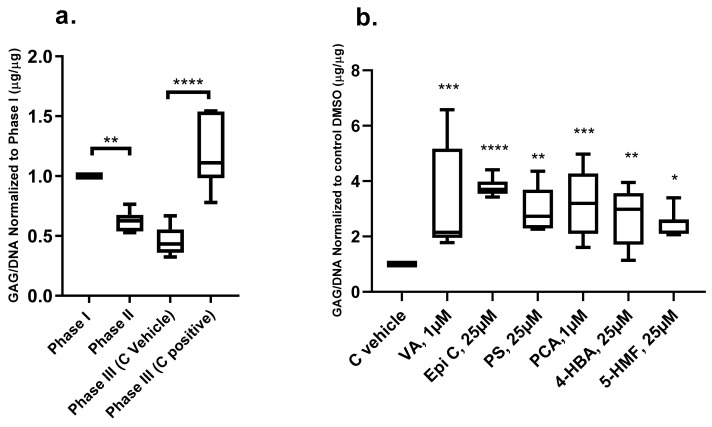
Biochemical analysis of the osteoarthritic chondrocyte pellets (long term) in the inflammatory model. **a**) GAG production per DNA in three different phases of the inflammatory model. **b**) The effect of TCM compounds on GAG/DNA ratio in *human* osteoarthritic chondrocytes in phase III after induction of inflammation normalized to control vehicle group (C vehicle). *n* = 3; n indicates the number of *human* OA chondrocytes donors; for each donor three experimental replicates were analyzed. For statistical analysis using Graphpad Prism, one-way analysis of variance (ANOVA) followed by Tukey’s post hoc test (multiple comparisons) for Figure 3a and Dunnett’s post hoc test (multiple comparisons) for Figure 3b were applied. * *p* < 0.01, ** *p* < 0.001, *** *p* < 0.0005, **** *p* < 0.0001.

After two weeks of cartilage generation with chondrogenic medium in phase I (long term) the cells produced significant amounts of matrix as assessed by dimethylmethylene blue (DMMB) assay and histology. After induction of inflammation with inflammatory cytokines in phase II (IL-1β/TNF-α), biochemical analysis showed that the GAG/DNA ratio was decreased compared with phase I (Figure 3a). In phase III, after two weeks of treatment (long term), the GAG/DNA ratio did not recover in the control vehicle group in which the cells were treated with chondro-permissive medium supplemented with 0.01%, 0.1%, 0.25% *v/v* DMSO. In the control positive group (chondrogenic medium) the GAG/DNA ratio reached a similar level as before induction of inflammation (Figure 3a). In the groups treated with VA, Epi C, PS, PCA, 4-HBA and 5-HMF in phase III, the amount of GAG production per DNA increased as compared to the control vehicle group (Figure 3b).

**Figure 4:**
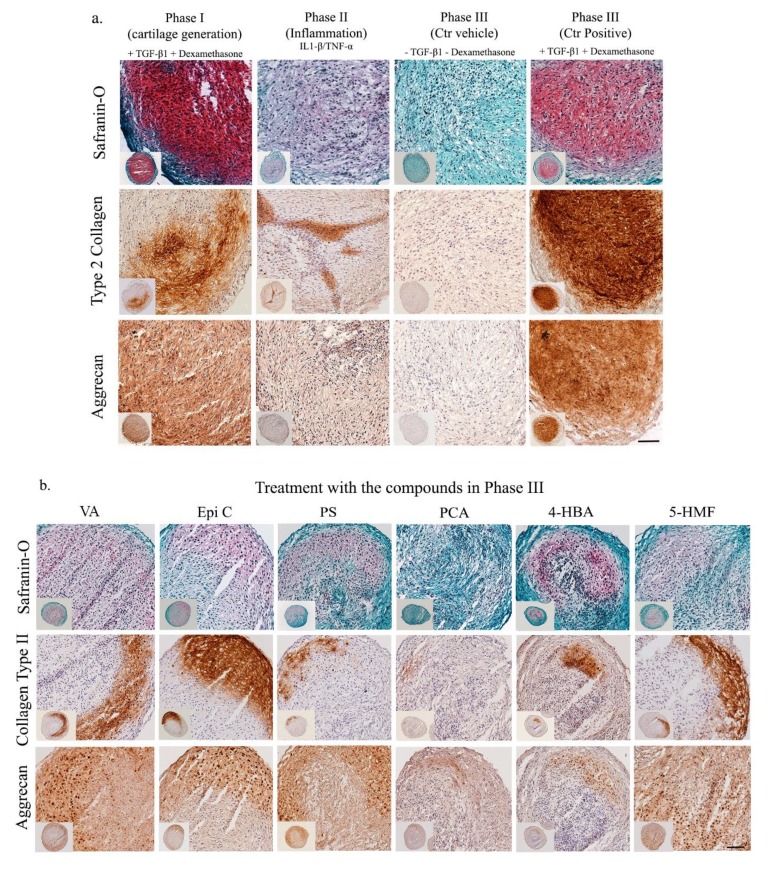
Histological and immunohistochemical characterization of pellets from OA chondrocytes in the inflammatory model (long term). **a**) Saf-O staining, COL-II and ACAN immunostaining of pellets in three different phases as the control groups (Phase I, Phase II, Phase III). **b**) and after 14 days of treatment with the TCM compounds in phase III (VA, Epi C, PS, PCA, 4-HBA, 5-HMF). Scale bars = 100 μm.

Histological analysis confirmed the observed biochemical results. In phase I, after two weeks of pellet culture in chondrogenic medium (long term), cartilage matrix was produced by osteoarthritic chondrocytes (Figure 4a). However, after induction of inflammation (phase II), the intensity of Safranin-O (Saf-O), COL-II and ACAN immunostaining significantly decreased. After two weeks of treatment (long term) in phase III, in the control positive group (chondrogenic medium), high intensity of Saf-O staining and immunostaining for COL-II and ACAN was observed, while staining for extracellular matrix (ECM) proteins in the control DMSO group was not noticeable (Figure 4a). After treatment with the compounds in the recovery phase III (long term), staining intensity for Saf-O, COL-II and ACAN in the groups treated with VA, Epi C, PS, PCA, 4-HBA and 5-HMF was preserved compared to phase II and stronger compared to the control vehicle group; though, the intensity of Saf-O staining and immunostaining for ECM proteins was still lower compared to phase I, and the compounds did not restore ECM to the same level as before induction of inflammation (Figure 4b).

## 3. Discussion

For an effective biological therapy against OA, it is essential to counteract elevated catabolism while increasing anabolism. Furthermore, it is necessary to inhibit pro-inflammatory cytokines that are excessively abundant in osteoarthritic joints [18]. In the current study, for the first time, the anabolic and anti-inflammatory effects of 34 bioactive compounds existing in the over-the-counter XLGB formula for the treatment of osteoporosis [32] and osteoarthritis [34] were assessed in 3D pellet culture using *human* OA chondrocytes. Osteoporosis is recognized as one of the most common bone loss conditions in which the elevated amount of pro-inflammatory cytokines can cause a systemic inflammatory condition [38,39,40,41]. Since OA is considered an inflammatory disease, we hypothesized that the compounds which could decrease bone resorption and stimulate new bone formation in postmenopausal osteoporosis, could also have the potential for treatment of OA. In fact, a recent clinical study showed both efficacy and safety of XLGB herbal formula for treatment of OA [34]. 

In our study, the cytotoxicity of the TCM compounds in monolayer culture was tested at four different concentrations (1 µM, 10 µM, 25 µM, 50 µM). Cell viability was more than 75% for all the compounds and even at the highest concentration no cytotoxicity was seen. In previous *in vitro* studies, compounds with positive chondrogenic and anti-inflammatory effects with an optimal dose between 1 µM and 50 µM have been reported [42,43,44,45]. In an in vivo study in adult *rabbits*, Icariin which was added to the cell-hydrogel constructs at a final concentration of 10 µM showed potential effect in cartilage tissue regeneration [36]. Furthermore, in a *rat* model of experimental OA, injection of 0.3 mL of 20 µM Icariin reduced the number of cartilage lesions and the expression of MMP-13 [46]. Since most of the tested compounds in our study are novel in the orthopedic field, the reported concentrations of representative compounds from each herbal extract (*Herba Epimedi*, *Radix ET Rhizoma Salviae, Fructus Psoraleae*) were considered [47,48,49,50,51]. Therefore, the anabolic effects of all the compounds in 3D pellet culture in chondropermissive medium (deprived of TGF-β and dexamethasone) and supplemented with compounds in four concentrations (1 µM, 10 µM, 25 µM, 50 µM) were tested. Nevertheless, we cannot exclude that lower or higher concentrations of certain compounds may have shown more pronounced effects. Additional studies will be required to identify the optimal dose for each single drug.

Since TGF-β and dexamethasone could mask the anabolic and anti-inflammatory effect of the compounds, these two essential chondrogenic components were removed from our culture media and replaced with TCM compounds. Among them, we reported bioactivity for Epi A, Epi C, 2-O-rhsII, Icariin, VA, PS, PCA, 4-HBA and 5-HMF, which could increase matrix production in *human* OA chondrocytes at different doses. Furthermore, at the transcriptional level in the inflammatory model, in pellets treated with Epi C, the expression of anabolic marker genes including *COL-II* and *ACAN* was significantly increased compared to the vehicle group. There is limited literature available on the anabolic effect of these compounds on articular chondrocytes. Icariin, as a widely-studied TCM compound with similar chemical structure as Epi C, has been proposed as a potential promoting compound for cartilage repair and can be used as a substitute for growth factors [36]. 

In contrast, the anti-inflammatory effects of certain TCM compounds are better characterized in the literature. Since IL-1β and TNF-α are key regulators of inflammatory pathways and induce the synthesis of MMPs, prostaglandins, aggrecanases [52] and other pro-inflammatory cytokines, these two cytokines were used to induce inflammation in our *in vitro* inflammatory model. Biochemical characterization of pellets (long term) demonstrated that adding 1 ng/mL of IL-1β and TNF-α significantly decreased extracellular matrix production of the samples. Although a certain donor variation was noticed, the same trend in three independent donors in response to inflammatory cytokines and after treatment with the compounds was observed. We showed that pellets (short term) treated with VA, Epi C, PS, PCA, 4-HBA and 5-HMF could reduce the inflammatory response, and expression of *MMP13*, the critical target gene in OA progression, was significantly inhibited [53,54]. In the groups treated with VA, Epi C, PCA, 4-HBA and 5-HMF, other catabolic marker genes including *MMP1* and *MMP3* were downregulated. Additionally, *COX-2* was inhibited in all the treatment groups except 4-HBA and PS. *COX-2* is recognized as an inflammatory enzyme which can promote other inflammatory mediators including prostaglandins. Inhibition of *COX-2* can provide relief from the symptoms of inflammation and pain [55]. The mechanism of action of acetylsalicylic acid (Aspirin^®^), the first NSAID, involves the inhibition of COX-2 and NFκB. Aspirin^®^ is developed from salicylates as the active components of natural Willow bark [56,57]. Interestingly, the chemical structures of VA, PCA and 4-HBA which are the extracts of *Radix ET Rhizoma Salviae*, is very similar to salicylates. Hence, these currently investigated small molecules with anti-inflammatory effects are likely to be active through hindering the NFκB signaling pathway. An in vivo study using a murine model of inflammatory pain showed that VA could inhibit pro-inflammatory cytokine production by suppressing NFκB activity [49], which supports our hypothesis on the mechanism of action of VA, PCA and 4-HBA. *O*-Vanilic acid, a derivative of VA, reduced pro-inflammatory cytokine levels by inhibiting LPS-induced degradation of IκBα and nuclear translocation of NFκB in LPS-stimulated macrophages [58]. It has been shown that low daily oral doses of 3- or 4-HBA in mice were acting like Aspirin^®^ and salicylic acids in terms of anti-inflammatory and analgesic effects [59]. PCA demonstrated promising anti-inflammatory and analgesic activity in *rat* models of both acute and chronic inflammation which was comparable with standard anti-inflammatory therapy [60]. Besides these compounds, we also reported Epi C and 5-HMF extracted from *Herba Epimedi*, as potential candidates with anti-inflammatory effects. It was demonstrated 5-HMF could suppress VCAM-1 expression in TNF-α stimulated HUVECs through inhibition of NFκB signaling pathway [45]. The anti-inflammatory effects of *Epimedium* plant extracts, such as Icariin, that act by inhibiting TNF-α and pro-inflammatory cytokines has been previously studied [61,62,63], and Epi C as a novel extract of this plant showed anti-inflammatory effects in our inflammatory model. Further study will be required to elucidate the exact signaling mechanisms of the compounds in *human* OA chondrocytes.

Histological and immunohistochemical staining showed that, after induction of inflammation by IL-1β/TNF-α, Saf-O staining and the staining intensity of ECM proteins, such as COL-II and ACAN, were significantly reduced. After treating pellets (long term) with VA, Epi C, PS, PCA, 4-HBA and 5-HMF, an increase in intensity for Saf-O, COL-II and ACAN in comparison with the control vehicle group was observed. These observations confirmed our inflammatory model, as well as previous results in which these compounds could preserve chondrogenic phenotype after induction of inflammation. After two weeks of treatment in phase III (recovery phase), the histological data of the control positive group treated with dexamethasone and TGF-β, showed that ECM matrix proteins including COL-II, ACAN and proteoglycans were significantly induced. However, it has been proved that in the presence of dexamethasone and TGF-β, the cells progress towards the hypertrophic phenotype [64]. Furthermore, an in vivo study in an osteoarthritic *mouse* model showed that the high concentration of active TGF-β1 in subchondral bone could initiate pathological changes leading to progression of OA, while inhibition of TGF-β1 activity in subchondral bone reduced the degradation of articular cartilage [65]. Since our compounds are less potent than TGF-β1, the parts of the pellets which were not exposed to the compound supplemented medium (due to culture in non-adherent v-bottom 96-well plates) demonstrated limited matrix production. To have an equal transport of drugs throughout the pellet, a trans-well system allowing all parts of the pellet to be simultaneously exposed to the compounds may be utilized. Additionally, in future studies, we will encapsulate the selected compound(s) in a hydrogel towards development of a local intra-articular delivery system.

In the current study, *human* OA chondrocytes were used, as they are the most clinically relevant source of cells for screening of potential OA treatments. However, due to use of *human* cells isolated from macroscopically evident OA regions of the joint, limited numbers of primary cells were obtained; therefore, passaged chondrocytes were used. Although, to minimize the cell de-differentiation an established expansion medium was used that promotes cell proliferation and maintains cell differentiation ability [66]. In further pre-clinical studies, the efficacy of the compounds will be validated in an osteochondral explant culture model using a cartilage bioreactor under mechanical stimulation [67] and finally tested in an osteoarthritic animal model. 

In summary, after screening of anabolic and anti-inflammatory effects of 34 bioactive compounds in different *in vitro* models of *human* OA chondrocytes, VA, Epi C, PS, PCA, 4-HBA and 5-HMF showed anti-inflammatory and anti-catabolic effects and could inhibit matrix degradation after induction of inflammation. Additionally, Epi C also induced an anabolic effect. These bioactive compounds, and combinations thereof, can be considered as anti-inflammatory and anabolic drugs for treatment of early OA. A local drug delivery system will be envisioned, as the permeability and bioavailability of the drugs in the joint are limited.

## 4. Materials and Methods

### 4.1. Drugs Screening on Human Osteoarthritic Chondrocytes

Thirty-four compounds (listed in Table 2) were investigated for their anabolic and/or anti-inflammatory effect (Chengdu Herbpurify Co.LTD and Chengdu Push Bio-Technology Co.LTD, Chengdu, China). The extraction method is shown in Supplementary Figure S1 [68]. All compounds were dissolved in dimethyl sulfoxide (DMSO) at a final concentration of 10 mg/mL and stored at 4 °C. Chondrocytes were treated with different concentrations of compounds (1 µM, 10 µM, 25 µM, 50 µM), as indicated in the specific experiments. These concentrations were selected from previous *in vitro* studies, where positive effects with doses between 1 µM and 50 µM had been reported [36,42].

### 4.2. Isolation of Human Osteoarthritic Chondrocytes and Cell Expansion

Cartilage tissue was obtained from three patients suffering from end stage OA and undergoing total knee arthroplasty (ages 47, 60 and 82 years; all female) at the university hospital of Basel under ethical agreement (Ethikkommission beider Basel, Ref.Nr. EK: 78/07, 20. March 2007). The cells were isolated as described elsewhere [69]. Briefly, tissue samples were minced with a scalpel into small pieces and were digested overnight in 0.2% collagenase II (300 U/mg, Worthington Biochemical Corp, Lakewood, NJ, USA) on an orbital shaker at 37 °C. Then, the isolated chondrocytes were expanded for three passages to 80% confluency in basal medium (BM, Dulbecco’s modified Eagle medium, high glucose (DMEM)), 10 mM HEPES, 1 mM sodium pyruvate, 1% penicillin/streptomycin (P/S), 100 μg/mL streptomycin, and 0.29 mg/mL glutamate (all from Gibco, Paisley, UK), supplemented with 10% fetal bovine serum (FBS), 5 ng/mL fibroblast growth factor-2 (FGF-2) and 1 ng/mL transforming growth factor (TGF)ß1 (both from Fitzgerald, Acton, MO, USA) in a humidified incubator (37 °C, 5% CO_2_).

### 4.3. Cell Toxicity Assay

Cytotoxicity of the compounds was assessed using the WST-1 reagent (Roche Applied Science, Mannheim, Germany) [70]. *Human* osteoarthritic chondrocytes were seeded in 96-well plates at a density of 2000 cells per well in 100 µL of DMEM containing 4.5 g/L glucose and supplemented with 5% FBS and 1% penicillin–streptomycin (P/S) (all products from Gibco). Cells were cultured at 37 °C, 5% CO_2_, 19% oxygen and 90% humidity. After 24 h of incubation, the cells were treated with TCM compounds in four different concentrations (1 µM, 10 µM, 25 µM, 50µM) or DMSO alone at concentrations of 0.01%, 0.1%, 0.25%, 0.5%, as control vehicle groups. After 48 h of treatment, medium was removed and 100 µL of a 10% *v/v* solution of WST-1 reagent in DMEM supplemented with 2.5% FBS was added to each well. After 4 h of incubation at 37 °C, absorbance of the samples was measured against a background control with a microplate reader (Victor 3, PerkinElmer, Waltham, MA, US) at 490 nm, with reference wavelength of 600 nm [71]. For this assay and further analyses, all treatment groups in different concentrations were normalized to the respective control vehicle group. Since DMSO in these concentrations (lower than 1%) did not show any significant effect on cells metabolism, normalization with the highest concentration of DMSO resulted in similar outputs [72]. 

### 4.4. Anabolic Effects of the TCM Compounds on Osteoarthritic Chondrocytes Pellet Cultures

Chondrogenic effect of TCM compounds was assessed by culturing the expanded chondrocytes at passage 3 in pellets. Standard BM (high glucose DMEM) was supplemented with 1.25 mg/mL *human* serum albumin (Gibco, Life Technologies Limited, Paisley, UK), ITS- Premix (Corning, Bedford, MA, USA), 0.1 mM ascorbic acid 2-phosphate (Sigma-Aldrich, St. Louis, MO, USA), and 1% P/S. Since the standard chondrogenic supplements TGF-β and dexamethasone could mask the anabolic effect of the compounds, these two essential components for chondrogenic differentiation were omitted from our culture medium (referred to as chondropermissive medium) and were replaced with the TCM compounds. Briefly, cells were re-suspended in chondropermissive medium as described before [73]. Aliquots of 1.5 × 10^5^ cells/150 μL were centrifuged at 400 g for 5 min in v-bottom non-adherent 96-well plates (Thermo Fisher scientific, Waltham, MA, US). Pellets were cultured for 2 weeks in a humidified incubator (37 °C, 5% CO_2_) with a change of medium twice a week. Medium was supplemented with TCM compounds in four different final concentrations (1 μM, 10 μM, 25 μM, 50 μM) or control vehicle group containing 0.01%, 0.1%, 0.25%, 0.5% *v/v* DMSO. 

### 4.5. Anti-Catabolic Effects of the TCM Compounds on Osteoarthritic Chondrocytes Pellet Cultures 

Passage 3 chondrocytes were centrifuged at 400 g for 5 min (2.5 × 10^5^ cells per pellet in 250 μL medium) in v-bottom, non-adherent 96-well plates and cultured in pellets using standard chondrogenic medium (chondropermissive medium containing 10 ng/mL TGFβ and 10^-7^ M dexamethasone (Sigma Aldrich)). After one week of culture in chondrogenic medium (short term), pellets were harvested for transcriptional analysis, and after two weeks of culture in chondrogenic medium (long term), pellets were harvested for biochemical and histochemical analysis (phase I). The remaining pellets were exposed to IL-1β and TNF-α (both from Peprotech, London, UK), each at 1 ng/mL, for 72 h (phase II), using an established inflammatory pellet culture model [74]. During this inflammatory phase, pellets were cultured in chondropermissive medium (chondrogenic medium deprived of TGF-β1 and dexamethasone) with or without TCM compound. Pellets were then harvested for analysis (phase II) or cultured two more weeks (phase III) in chondropermissive medium supplemented with TCM compounds or DMSO as control vehicle group. The most potent compounds in terms of their anabolic effects were selected for testing in this inflammatory model, in 3 different concentrations (1 μM, 10 μM, 25 μM). The positive control (ctr +) group was supplemented with 10 ng/mL TGF-β1 and 10^-7^ M dexamethasone (phase III). Cell pellets were maintained at 37 °C, 5% CO_2_, with medium changes twice per week. The different experimental groups are summarized in Table 3. 

### 4.6. Biochemical Analysis

Pellets (*n* = 3) from three donors were digested with 0.5 mL of 1 mg/mL proteinase K at 56 °C for 16 h (Roche, Mannheim, Germany). Using 1,9 dimethylmethylene blue (DMMB) assay, the amount of sulfated glycosaminoglycan (GAG) within the pellets was measured spectrophotometrically with chondroitin sulphate as a standard (Sigma-Aldrich) [75]. The DNA content was measured spectrofluorometrically using PicoGreen (Invitrogene, Eugene, OR, USA) dye with calf thymus DNA (LuBio Science, Zurich, Switzerland) as a standard and according to the manufacturer’s instruction. The GAG content was normalized to the DNA content in all samples. 

### 4.7. Gene Expression Analysis

For total RNA extraction, 1 mL of TRI reagent (Molecular Research Center) was added to the pellet. After homogenization by a Tissue Lyser for 3 min and 5 Hz (Qiagen, Hilden, Germany), the RNA was extracted using phase separation by 1-Bromo-3-chloropropane (Sigma) in a volume ratio of 1:10 with the TRI reagent. Reverse transcription was performed with SuperScript VILO cDNA Synthesis Kit (Life Technologies, Carlsbad, CA). Quantitative real-time PCR (qPCR) was accomplished using TaqMan Universal Master Mix (Applied biosystems, Foster City, CA, USA) and the Quant Studio 6 Flex Instrument and software (Applied biosystems) were used for detection. The gene expression assays and sequences of *human* primers and TaqMan probes for *human 18S rRNA*, *MMP1, MMP3, MMP13, COX-2*, *COL-II* and *ACAN* are shown in Table 4 (a, b). The relative gene expression was calculated using the 2^-ΔΔCT^ quantification method [76], with 18S ribosomal RNA as endogenous control.

### 4.8. Histological and Immunohistochemical Analysis

Pellets were fixed in 70% methanol and incubated overnight in 5% sucrose solution at 4 °C. Then, the samples were embedded in cryo-compound and were sectioned with cryostat at 8 µm thickness. To visualize cells, proteoglycan and the collagen deposition, slides were first stained with Weigert’s Haematoxylin for 10 min. Sections were stained with 0.02% Fast green in ultrapure (ddH2O) water for 5 min to reveal collagen deposition, then stained with 0.1% Saf-O for 12 min to show proteoglycan deposition, and finally rinsed in dH_2_O and sequentially differentiated in 70%, 96% and 100% ethanol. The presence of ACAN and COL-II was identified immunohistochemically using antibodies against ACAN (1-C-6) and COL-II (CIIC1), both from Developmental Studies Hybridoma Bank, University of Iowa). Briefly, after enzyme treatment with hyaluronidase (Sigma-Aldrich, St. Louse, MO, USA) for COL-II and chondroitinase (Sigma-Aldrich) for 1C6, non-specific binding sites were blocked with horse serum (Vector Laboratories, Burlingame, CA, USA) for 1h at RT. The primary antibody (5 µg/mL) was incubated for 30 min and detected using secondary biotinylated anti-*mouse* antibody (Vector Laboratories) followed by incubation with avidin-biotin-peroxidase complex (Vectastain ABC Kit, *Mouse* IgG, Vector Laboratories). Peroxidase activity was visualized using diaminobenzidine (DAB) as a substrate (ImmPACT DAB, Substrate for Peroxidase, Vector Laboratories) [77].

### 4.9. Statistical Analysis

Statistical comparisons were performed using Graphpad Prism 7. One-way analysis of variance (ANOVA) followed by Dunnett’s post hoc test (multiple comparison) or Tukey’s post hoc test (multiple comparison) was applied as non-parametric test of three independent experiments with different *human* osteoarthritic chondrocytes. To assess statistical significance among the groups, differences were considered statistically significant at *p* < 0.05. All Graphs are displayed as box plots. 

## Figures and Tables

**Table 1:** Relative viable cell count after treatment with the 34 compounds measured by WST-1 assay.

Compound	% Cell Numbers (Normalized to Control Vehicle)				
	Conc.[µM]	1	10	25	50
5-Hydroxymethylfurfural		111.4 ± 6.6	107.0 ± 11.2	110.2 ± 9.4	107.9 ± 3.3
Protocatechuicaldehyde		100.1 ± 4.2	96.0 ± 9.3	94.8 ± 6.4	81.2 ± 5.4
Vanilic acid		102.1 ± 2.4	96.8 ± 3.7	115.7 ± 3.2	112.6 ± 2.1
4-Hydroxybenzoic acid		103.4 ± 2.5	102 ± 9.6	105 ± 3.4	78.6 ± 6.9
Chlorogenic acid		77.8 ± 3.2	78.6 ± 4.8	77.8 ± 5.2	79.8 ± 5.6
Cryptochlorogenic acid		86.2 ± 8.5	86 ± 7.9	93.8 ± 5.2	78.3 ± 8.4
Loganic acid		85.6 ± 3.6	94.7 ± 3	91.5 ± 1.7	93.6 ± 4.9
Loganin		107 ± 5.6	109.2 ± 5.1	110.6 ± 9.8	102.8 ± 3.6
Isobavachalcone		102.1 ± 6.6	108.7 ± 5.7	105.7 ± 0.6	88.4 ± 8.8
Sweroside		110.2 ± 2.3	106.7 ± 4.1	112.5 ± 1.8	107.7 ± 9.8
(+)-Cycloolivil		101.5 ± 1.5	98. ± 2.1	105.6 ± 4.9	103.2 ± 8.2
Baohuoside I		82.8 ± 6.3	76.3 ± 1.8	77.5 ± 0.4	79.8 ± 5.8
2’-O-rhamnosylicariside II		102.1 ± 6.6	105.5 ± 4.7	91.8 ± 3.2	102.9 ± 2.1
Epimedin A		83.9 ± 5.6	84.8 ± 8.1	97.1 ± 6.9	111.3 ± 5.1
Epimedin B		82.7 ± 9.2	88 ± 9.9	87.8 ± 9.1	94. ± 7.9
Epimedin C		87.5 ± 0.6	89.5 ± 6.5	88.1 ± 3.7	89.8 ± 6.4
Isobavachin		87.3 ± 1.3	91.5 ± 8.2	89.2 ± 0.8	85.7 ± 0.1
Bavachin		109.1 ± 2.1	102 ± 8.4	89.2 ± 9.5	91.7 ± 2.1
Bavachinin		96.4 ± 1.1	78.1 ± 6.6	79.9 ± 5.1	79.7 ± 3
Neobavaisoflavone		84.4 ± 8.6	89.6 ± 1.9	99 ± 2.5	76.1 ± 2.2
Corylin		89.6 ± 2.4	94 ± 3.7	87.8 ± 1.6	79.3 ± 3.1
Epimedin A1		95.48 ± 4.3	85.49 ± 0.5	88.21 ± 3.1	95.46 ± 2.4
Psoralen		83.6 ± 5.1	80.3 ± 5.3	85.1 ± 6.4	84.8 ± 8.3
Isopsoralen		86.2 ± 8.6	80.2 ± 1.9	83 ± 2.7	85.4 ± 3.2
(S)-Bukuchiol		89.1 ± 4.4	94.4 ± 3.5	104.7 ± 4.2	88.4 ± 4.2
Psoralidin		106.5 ± 6.3	84.7 ± 1.4	86.4 ± 5.9	85.1 ± 4.8
Asperosaponin VI		93.9 ± 2.8	99 ± 9.2	103.8 ± 4.5	93.9 ± 4.8
Baohuoside II		103.6 ± 5.9	96.4 ± 7.6	95 ± 5.2	79.7 ± 2.9
Epimedoside A		89 ± 4.7	87.9 ± 3.1	90.6 ± 4.4	85.6 ± 3.7
Baohuoside V		98.6 ± 8.7	100 ± 6.5	100.9 ± 7.8	78.5 ± 3.8
Corylifol A		86.1 ± 3.3	85.3 ± 1.8	94.6 ± 0.9	103.8 ± 2.6
4’-O-Methyl-broussochalcone		81.4 ± 2.9	85.4 ± 2.4	95.4 ± 3.6	97.5 ± 2.3
Anhydroicaritin		85.2 ± 2.2	83.4 ± 6.6	86.2 ± 1.5	81.8 ± 2.4
Icariin		95.7 ± 3.6	101.4 ± 3.7	95.5 ± 3.2	101.5 ± 5.7

The compounds were tested in different concentrations (1 µM, 10 µM, 25 µM, 50 µM) for 48 h. Data is normalized to the control vehicle group (cells with medium and DMSO).

**Table 2:** List of 34 tested Traditional Chinese Medicine (TCM) compounds extracted from XLGB with their chemical formula, molecular weight and component herb.

No	Name of the Compound	Molecular Formula	Molecular Weight	Component Herb
1	5-Hydroxymethylfurfural	C_6_H_6_O_3_	126	H.E
2	Protocatechuicaldehyde	C_7_H_6_O_3_	138	R.S
3	Vanilic acid	C_8_H_8_O_4_	168	R.S
4	4-Hydroxybenzoic acid	C_7_H_6_O_3_	138	R.S
5	Chlorogenic acid	C_16_H_18_O_9_	354	H.E
6	Cryptochlorogenic acid	C_16_H_18_O_9_	354	H.E
7	Loganic acid	C_16_H_24_O_10_	376	R.D
8	Loganin	C_17_H_26_O_10_	390	R.D
9	Isobavachalcone	C_20_H_20_O_4_	324	F.P
10	Sweroside	C_16_H_22_O_9_	358	R.D
11	(+)-Cycloolivil	C_20_H_24_O_7_	376	H.E
12	Baohuside I	C_27_H_30_O_10_	514	H.E
13	2′′-*O*-rhamnosylicariside II	C_33_H_40_O_14_	660	H.E
14	Epimedin A	C_39_H_50_O_2_	838	H.E
15	Epimedin B	C_38_H_48_O_19_	808	H.E
16	Epimedin C	C_39_H_50_O_19_	822	H.E
17	Isobavachin	C_20_H_20_O_4_	324	F.P
18	Bavachin	C_20_H_20_O_4_	324	F.P
19	Bavachinin	C_21_H_22_O_4_	338	F.P
20	Neobavaisoflavone	C_20_H_18_O_4_	322	F.P
21	Corylin	C_20_H_16_O_4_	320	F.P
22	Epimedin A1	C_39_H_50_O_20_	838	H.E
23	Psoralen	C_11_H_6_O_3_	186	F.P
24	Isopsoralen	C_11_H_6_O_3_	186	F.P
25	(*S*)-Bukuchiol	C_18_H_24_O	256	F.P
26	Psoralidin	C_20_H_16_O_5_	336	F.P
27	Asperosaponin VI	C_47_H_76_O_18_	390	R.D
28	Baohuside II	C_26_H_28_O_10_	500	H.E
29	Epimedoside A	C_32_H_38_O_15_	662	H.E
30	Baohuside V	C_39_H_50_O_19_	822	H.E
31	Corylifol A	C_25_H_26_O_4_	390	F.P
32	4′-Methylbavachalcone	C_21_H_22_O_4_	338	F.P
33	Icaitin	C_21_H_20_O_6_	368	H.E
34	Icariin	C_33_H_40_O_15_	676	H.E

H.E.: Herba Epimedi; R.S.: Radix ET Rhizoma Salviae; R.D.: Radix Dipsaci; F.P.: Fructus Psoraleae.

**Table 3:** Description of experimental groups.

Pellet Culture	Group	Culture Phase I (Cartilage Generation)	Culture Phase II (IL-1β/TNFα Exposure; Inflammatory phase)		Culture Phase III (Treatment)	
		Time	Time	Compound	Time	Compound
	Control (IL-1β/TNFα)	1 week	3 days	--	n/a	
Short term	Treatment group (IL-1β/TNFα + compound)	1 week	3 days	+	n/a	
	Control (IL-1β/TNFα)	2 weeks	3 days	--	2 weeks	--
Long term	Treatment group (IL-1β/TNFα - compound)	2 weeks	3 days	+	2 weeks	+

**Table:** a. Primers and probes (Applied Biosystems).

Gene	Probe Type	Assay ID
*MMP-1*	5′ FAM-3′ NFQ	Hs00899658_m1
*MMP-3*	5′ FAM-3′ NFQ	Hs00968305_m1
*18s fast*	5′ FAM-3′ NFQ	Hs99999901_s1

*MMP1*: matrix metalloproteinase 1; *MMP3*: matrix metalloproteinase 3; FAM: Carboxyfluorescein; NFQ: nonfluorescent quencher a. *Human* Gene Expression Assays (ThemoFisher Scientific). b.

**Table:** b. Custom Designed Primer/Probe (Microsynth, Balgach, Switzerland).

Gene	Primer/Probe Type	Sequence
*MMP-13*	Primer forward (5′-3′) Primer reverse (5′-3′) Probe (5′ FAM/3′ TAMRA)	CGGCCACTCCTTAGGTCTTG TTTTGCCGGTGTAGGTGTAGATAG CTCCAAGGACCCTGGAGCACTCATGT
*COX-2*	Primer forward (5′-3′) Primer reverse (5′-3′) Probe (5′ FAM/3′ TAMRA)	TTGTACCCGGACAGGATTCTATG TGTTTGGAGTGGGTTTCAGAAATA GAAAACTGCTCAACACCGGAATTTTTGACAA
*Col2a1*	Primer forward (5′-3′) Primer reverse (5′-3′) Probe (5′ FAM/3′ TAMRA)	GGCAATAGCAGGTTCACGTACA GATAACAGTCTTGCCCCACTTACC CCTGAAGGATGGCTGCACGAAACATAC
*ACAN*	Primer forward (5′-3′) Primer reverse (5′-3′) Probe (5′ FAM/3′ TAMRA)	AGTCCTCAAGCCTCCTGTACTCA CGGGAAGTGGCGGTAACA CCGGAATGGAAACGTGAATCAGAATCAACT

*COL2A1*: collagen type II; *MMP13*: matrix metalloproteinase 13; *COX-2*: cyclooxygenase-2; *ACAN:* aggrecan; FAM: Carboxyfluorescein; TAMRA: Tetramethylrhodamine.

## Supplementary Materials

Supplementary materials can be found at https://www.mdpi.com/1422-0067/20/22/5745/s1.
