# Supplementary material for: Regulation of Inflammatory Response in Human Osteoarthritic Chondrocytes by Novel Herbal Small Molecules
Source: Int J Mol Sci. 2019 Nov 15;20(22):5745. doi: 10.3390/ijms20225745 (PMC6888688; doi:10.3390/ijms20225745)
Supplement: Supplementary file 1 [file ijms-20-05745-s001.zip › suplemantary table 1.pdf]

Compound	GAG/DNA ratio in three human OA chondrocytes donors				
	Conc.[μM]	1	10	25	50
**5-Hydroxymethylfurfural**		2.07 ± 0.43	2.08 ± 0.26	2.79 ± 0.62	2.62 ± 0.36
**Protocatechuicaldehyde**		3.39±1.23	2.97 ± 0.66	2.23 ± 0.47	2.05 ± 0.32
**Vanillic acid**		3.47 ± 0.12	2.58 ± 0.65	3.32 ± 1.22	2.14 ± 0.41
**4-Hydroxybenzoicacid**		2.01 ± 0.65	1.98 ± 0.33	2.89 ± 0.53	1.34 ± 0.63
**Chlorogenic acid**		1.53 ± 0.23	2.52 ± 0.97	2.07 ± 0.32	0.88 ± 0.39
**Cryptochlorogenic acid**		2.26 ± 0.55	1.29 ± 0.42	2.74 ± 0.87	0.91 ± 0.21
**Loganic acid**		0.85 ± 0.13	0.91 ± 0.26	0.82 ± 0.58	0.93 ± 0.22
**Loganin**		0.71 ± 0.14	1.69 ± 0.42	2.31 ± 0.37	0.16 ± 0.22
**Isobavachalcone**		1.70 ± 0.37	2.30 ± 0.75	1.59 ± 0.62	1.16 ± 0.56
**Sweroside**		2.17 ± 0.33	1.83 ± 0.82	2.07 ± 0.94	1.35 ± 0.22
**(+)-Cycloolivil**		2.49 ± 0.52	1.18 ± 0.58	1.82 ± 0.32	0.19 ± 0.07
**Baohuoside I**		2.54 ± 0.68	2.92 ± 0.42	1.65 ± 0.74	0.59 ± 0.04
**2'-O-rhamnosylicariside II**		3.40 ± 0.65	2.48 ± 0.53	3.65 ± 0.42	2.46 ± 0.23
**Epimedin A**		2.92 ± 0.64	2.54 ± 0.23	3.64 ± 0.35	2.18 ± 0.48
**Epimedin B**		2.14 ± 0.46	2.04 ± 0.22	2.75 ± 0.74	1.62 ± 0.26
**Epimedin C**		2.16 ± 0.54	2.27 ± 0.30	3.82 ± 0.43	3.53 ± 0.32
**Isobavachin**		1.74 ± 0.64	2.00 ± 0.28	1.50 ± 0.31	1.18 ± 0.46
**Bavachin**		2.21 ± 0.34	2.05 ± 0.63	2.38 ± 0.83	0.84 ± 0.13
**Bavachinin**		0.12 ± 0.07	0.32 ± 0.13	0.24 ± 0.02	0.17 ± 0.03
**Neobavaisoflavone**		1.08 ± 0.12	0.85 ± 0.14	0.91 ± 0.29	0.14 ± 0.05
**Corylin**		0.59 ± 0.15	0.88 ± 0.12	0.90 ± 0.43	0.53 ± 0.16
**Epimedin A1**		1.03 ± 0.32	0.88 ± 0.57	0.92 ± 0.14	0.08 ± 0.04
**Psoralen**		1.47 ± 0.48	1.31 ± 0.34	1.70 ± 0.63	0.81 ± 0.38
**Isopsoralen**		1.15 ± 0.47	0.90 ± 0.29	0.80 ± 0.14	0.84 ± 0.22
**(S)-Bukuchiol**		1.13 ± 0.35	1.15 ± 0.42	0.79 ± 0.26	0.88 ± 0.12
**Psoralidin**		2.18 ± 0.57	1.67 ± 0.42	2.56 ± 0.78	1.74 ± 0.85
**Asperosaponin VI**		0.83 ± 0.18	0.80 ± 0.23	0.79 ± 0.19	0.79 ± 0.34
**Baohuoside II**		0.59 ± 0.03	0.79 ± 0.24	1.04 ± 0.28	0.77 ± 0.09
**Epimedoside A**		0.93 ± 0.07	0.82 ± 0.14	0.82 ± 0.04	0.67 ± 0.17
**Baohuoside V**		0.96 ± 0.08	0.92 ± 0.05	0.88 ± 0.06	0.76 ± 0.03
**Corylifol A**		1.14 ± 0.42	1.27 ± 0.31	1.03 ± 0.03	0.64 ± 0.07
**4'-O-Methyl-broussochalcone**		0.95 ± 0.22	0.78 ± 0.14	0.89 ± 0.19	0.89 ± 0.23
**Anhydroicaritin**		0.93 ± 0.02	0.80 ± 0.26	1.36 ± 0.28	0.93 ± 0.14
**Icariin**		2.28 ± 0.17	2.25 ± 0.32	2.97 ± 0.53	2.81 ± 0.52

Supplementary Table 1. Anabolic effects of TCM compounds on glycosaminoglycan production
