# Supplementary material for: Regulation of Inflammatory Response in Human Osteoarthritic Chondrocytes by Novel Herbal Small Molecules
Source: Int J Mol Sci. 2019 Nov 15;20(22):5745. doi: 10.3390/ijms20225745 (PMC6888688; doi:10.3390/ijms20225745)
Supplement: Supplementary file 1 [file ijms-20-05745-s001.zip › Supplemantory figure 1.pdf]

1. Isolation procedure for "Xian-Ling-Gu-Bao" extraction

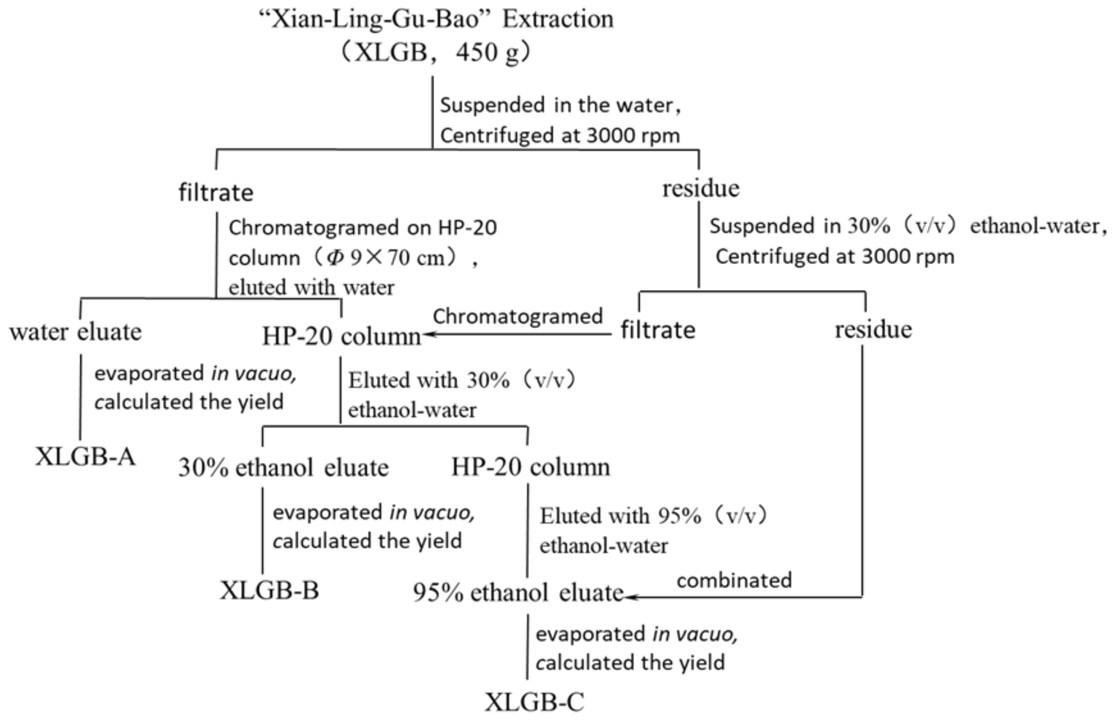

2. Isolation procedure for active fractions XLGB-B and XLGB-C

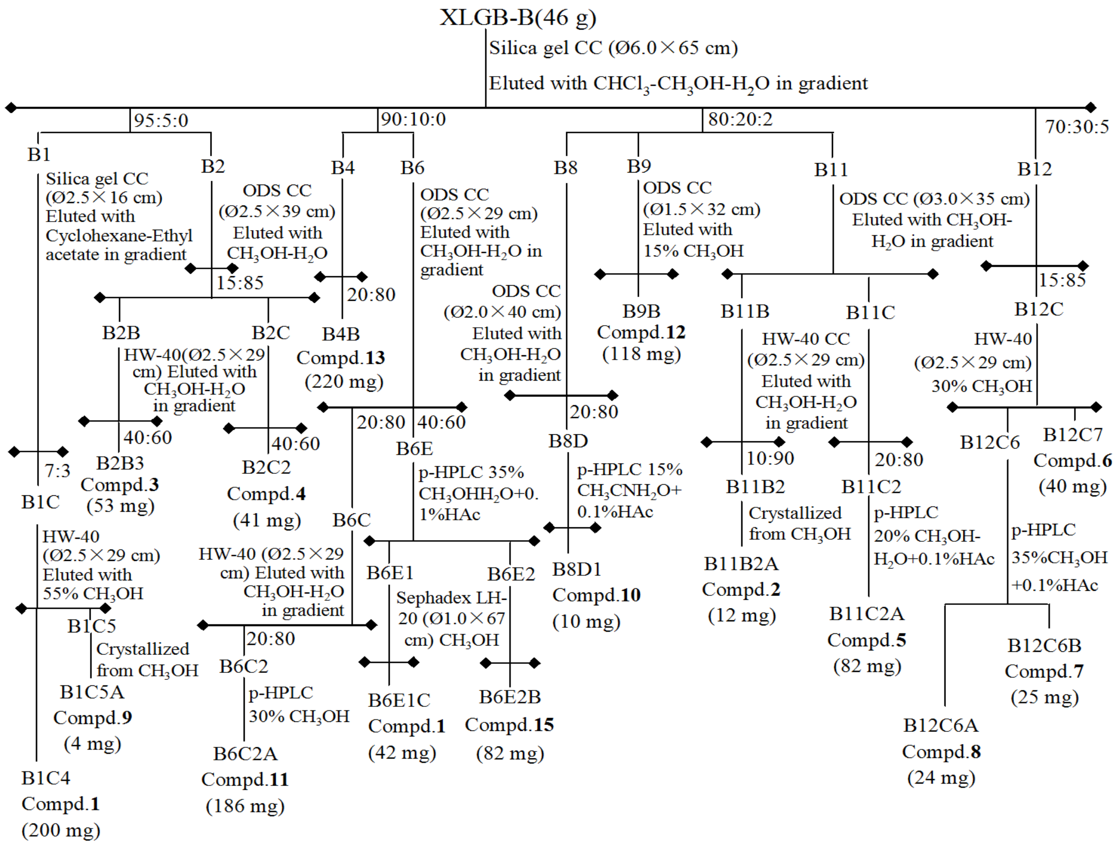

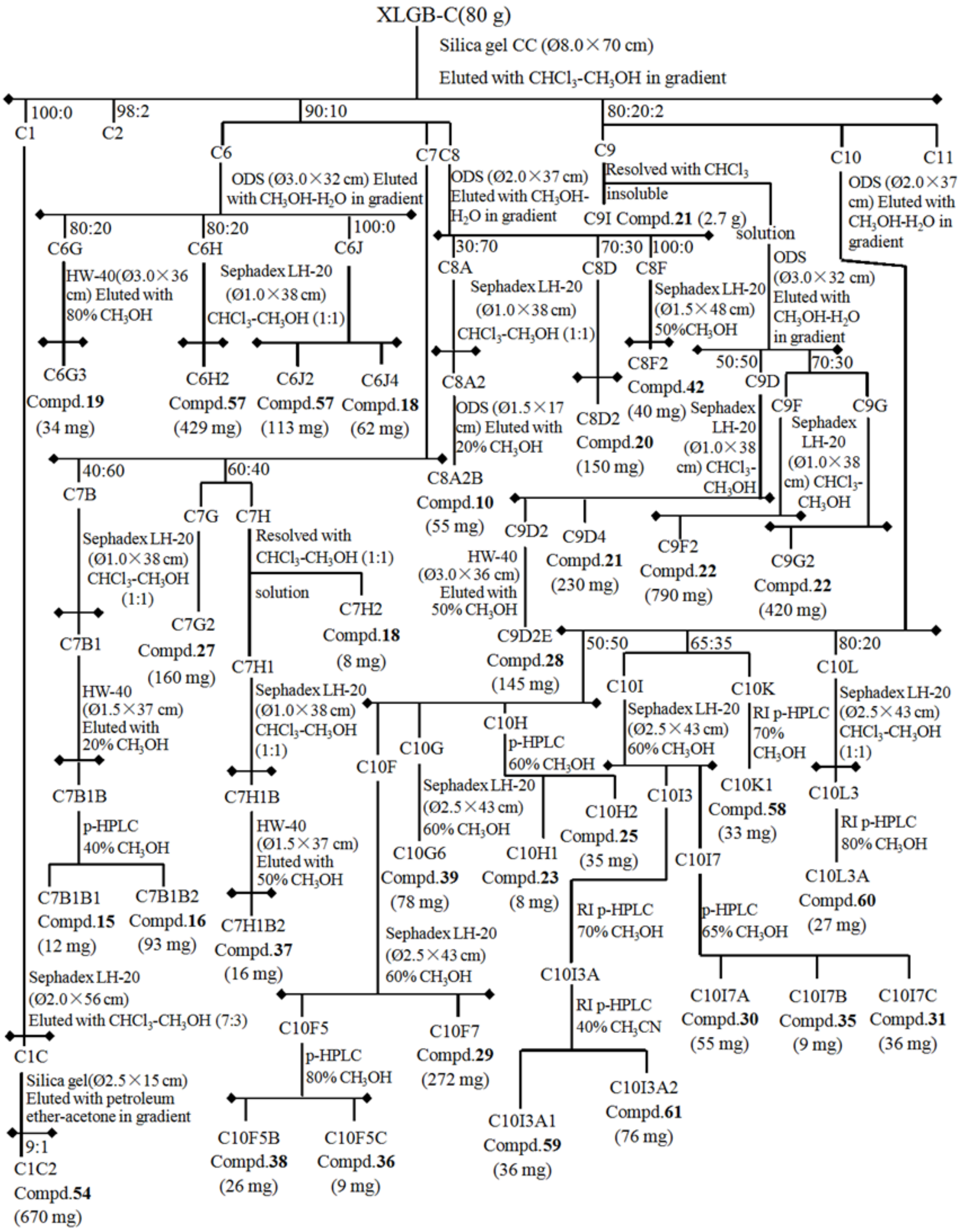

XLGB-C(80 g)
Silica gel CC (Ø8.0×70 cm)
Eluted with CHCl3-CH3OH in gradient
100:0
98:2
90:10
80:20:2
C1
C2
C6
C7C8
C9
C10
C11
ODS (Ø3.0×32 cm) Eluted with CH3OH-H2O in gradient
ODS (Ø2.0×37 cm) Eluted with CH3OH-H2O in gradient
Resolved with CHCl3
insoluble
C9I Compd.21 (2.7 g)
solution
ODS (Ø2.0×37 cm) Eluted with CH3OH-H2O in gradient
80:20
C6G
80:20
C6H
100:0
C6J
30:70
C8A
70:30
C8D
100:0
C8F
HW-40(Ø3.0×36 cm) Eluted with 80% CH3OH
Sephadex LH-20 (Ø1.0×38 cm) CHCl3-CH3OH (1:1)
Sephadex LH-20 (Ø1.0×38 cm) CHCl3-CH3OH (1:1)
Sephadex LH-20 (Ø1.5×48 cm) 50%CH3OH
ODS (Ø3.0×32 cm) Eluted with CH3OH-H2O in gradient
C6G3 Compd.19 (34 mg)
C6H2 Compd.57 (429 mg)
C6J2 Compd.57 (113 mg)
C6J4 Compd.18 (62 mg)
C8A2
C8F2 Compd.42 (40 mg)
50:50
C9D
70:30
C9F
C9G
ODS (Ø1.5×17 cm) Eluted with 20% CH3OH
C8D2 Compd.20 (150 mg)
Sephadex LH-20 (Ø1.0×38 cm) CHCl3-CH3OH
Sephadex LH-20 (Ø1.0×38 cm) CHCl3-CH3OH
40:60
C7B
60:40
C7G
C7H
C8A2B Compd.10 (55 mg)
C9D2
C9D4 Compd.21 (230 mg)
C9F2 Compd.22 (790 mg)
C9G2 Compd.22 (420 mg)
Sephadex LH-20 (Ø1.0×38 cm) CHCl3-CH3OH (1:1)
Resolved with CHCl3-CH3OH (1:1)
solution
C7H2 Compd.18 (8 mg)
HW-40 (Ø3.0×36 cm) Eluted with 50% CH3OH
C9D2E Compd.28 (145 mg)
C7G2 Compd.27 (160 mg)
C7B1
C7H1
50:50
65:35
80:20
C10I
C10K
C10L
HW-40 (Ø1.5×37 cm) Eluted with 20% CH3OH
Sephadex LH-20 (Ø1.0×38 cm) CHCl3-CH3OH (1:1)
C10F
C10G
C10H
p-HPLC 60% CH3OH
Sephadex LH-20 (Ø2.5×43 cm) 60% CH3OH
RI p-HPLC 70% CH3OH
Sephadex LH-20 (Ø2.5×43 cm) CHCl3-CH3OH (1:1)
C7B1B
C7H1B
Sephadex LH-20 (Ø2.5×43 cm) 60% CH3OH
C10H2 Compd.25 (35 mg)
C10I3
C10K1 Compd.58 (33 mg)
C10L3
p-HPLC 40% CH3OH
HW-40 (Ø1.5×37 cm) Eluted with 50% CH3OH
C10G6 Compd.39 (78 mg)
C10H1 Compd.23 (8 mg)
C10I7
RI p-HPLC 80% CH3OH
C10L3A Compd.60 (27 mg)
C7B1B1 Compd.15 (12 mg)
C7B1B2 Compd.16 (93 mg)
C7H1B2 Compd.37 (16 mg)
RI p-HPLC 70% CH3OH
p-HPLC 65% CH3OH
Sephadex LH-20 (Ø2.5×43 cm) 60% CH3OH
C10I3A
Sephadex LH-20 (Ø2.0×56 cm) Eluted with CHCl3-CH3OH (7:3)
C10I7A Compd.30 (55 mg)
C10I7B Compd.35 (9 mg)
C10I7C Compd.31 (36 mg)
C1C
C10F5
C10F7 Compd.29 (272 mg)
RI p-HPLC 40% CH3CN
Silica gel(Ø2.5×15 cm) Eluted with petroleum ether-acetone in gradient
p-HPLC 80% CH3OH
C10I3A2 Compd.61 (76 mg)
9:1
C10I3A1 Compd.59 (36 mg)
C1C2 Compd.54 (670 mg)
C10F5B Compd.38 (26 mg)
C10F5C Compd.36 (9 mg)
